# ﻿*Entyposisfrici* (Coleoptera, Scarabaeidae, Melolonthinae), a new species from Somaliland

**DOI:** 10.3897/zookeys.1165.101908

**Published:** 2023-05-30

**Authors:** Aleš Bezděk, Richard Sehnal, Hassan S. A. Elmi, David Sommer, David Král

**Affiliations:** 1 Biology Centre of the Czech Academy of Sciences, Institute of Entomology, Branišovská 31, CZ-370 05 České Budějovice, Czech Republic Biology Centre of the Czech Academy of Sciences, Institute of Entomology České Budějovice Czech Republic; 2 Czech University of Life Sciences Prague, Faculty of Agrobiology, Food and Natural Resources, Department of Zoology and Fisheries, Kamýcká 129, CZ-165 21 Praha 6 – Suchdol, Czech Republic Czech University of Life Sciences Prague Praha Czech Republic; 3 Department of Biology, Faculty of Education, Amoud University, Borama 25263, Somalia Amoud University Borama Somalia; 4 Department of Zoology, Faculty of Science, Charles University, Viničná 7, CZ-128 00 Praha 2, Czech Republic Charles University Praha Czech Republic

**Keywords:** Description, Ethiopian region, Horn of Africa, Scarabaeoidea, Schizonychini, taxonomy

## Abstract

*Entyposisfrici* Bezděk & Sehnal, **sp. nov.**, from Somaliland is described and relevant diagnostic characters are illustrated. The new species is compared with the morphologically closely similar *Entyposis* Kolbe, 1894 species. An updated checklist and an identification key to northeastern African *Entyposis* species are provided.

## ﻿Introduction

The genus *Entyposis* Kolbe, 1894 forms a characteristic group with striking sexual dimorphism within the tribe Schizonychini (Coleoptera, Scarabaeidae, Melolonthinae). The pronotum of males is thickened basally into conspicuous bulges and has a deep medial impression that widens anteriorly into a prominent tubercle. Both the basal bulges and the medial impression are only weakly developed in females. The rather similar genus *Entypophana* Moser, 1913 differs from *Entyposis* primarily in the shape of the occipital carina, which is simple in *Entyposis* but elevated medially into a broad edge or simple (occasionally double) horn in *Entypophana* (Lacroix and Montreuil 2012; Sehnal 2017).

According to a recent revision of *Entyposis* by Lacroix and Montreuil (2012), there were nine species distributed in eastern Africa. Thenceforth, two more papers have been published. Sehnal (2017) described a new *Entyposis* from southern Ethiopia, and later he (Sehnal 2019) synonymized *Proseconius* Kolbe, 1894 with *Entyposis* and *E.cavicollis* (Fairmaire, 1887) with *P.capito* (Gerstaecker, 1873). Thus, 10 *Entyposis* species are known to be distributed from southern Ethiopia to northern Zimbabwe.

Within the rich material recently collected by Czech entomologists in Somaliland during 2021–2022, we found a pair (male and female) of *Entyposis* new to science, the description of which is presented below. The presence of this new Somaliland species significantly extends the range of *Entyposis* in a northeasterly direction towards the Horn of Africa.

## ﻿Materials and methods

The specimens were examined with a Novex stereomicroscope; measurements were taken with an ocular grid. Length measurements are from the anterior margin of the clypeus to the apex of the elytron. The habitus photographs were taken with a Canon MP-E 65mm/2.8 1–5× macro lens attached to a Canon EOS 90D camera. Partially focused images of each specimen were stacked using the Helicon Focus v. 3.20.2 Pro software.

Specimens in the type series are provided with one red printed label: “*Entyposisfrici* sp. nov., HOLOTYPUS or PARATYPUS [with sex symbol], A. Bezděk and R. Sehnal det. 2023”. Verbatim label data are cited for type material examined. Lines within each label are separated by a vertical slash [|]. Information in quotes indicates the original spelling. Authors’ remarks and additional comments are placed in brackets [].

The following codes identify the collections housing the material examined (curators in round brackets):

**BMNH**Natural History Museum, London, United Kingdom (Maxwell V.L. Barclay, Michael Geiser, Keita Matsumoto);

**NMPC**Natural History Museum, London, United Kingdom (Jiří Hájek);

**RSCV** Richard Sehnal collection, Velenice, Czech Republic.

All fieldwork in this study complied with legal Somaliland regulations and sampling was in accordance with local legislation (export permit Ref. MOERD/M/I/251/2021).

Material used for comparison:

*Entyposiscordipenis* Sehnal, 2017. 8 males and 3 females, all paratypes (RSCV, Figs 3, 4, 11, 12, 19, 20, 27, 28, 34, 38): “ETHIOPIA – Hamer or. Turmi near 950 m. | 04°58'31"N 036°30'53"E | 27–30.11.2016 | Vladimír Major leg.”; Ethiopia • 4 males (RSCV), Turmi, Mango Lodge, 920 m, 25.–29.XI.2015, leg. Ströhe.

*Entyposisimpressa* Kolbe, 1894. Kenya • 1 male (RSCV), Shanzu, Mailika, 10.IV.2002 (Figs 5, 13, 21, 29, 35, 39); 2 males and 10 females (BMNH), Garissa Bura, Tana riv., IX.[19]48, leg. van Someren (Figs 6, 14, 22, 30); 1 female (BMNH), Garissa Bura, Ukazzi hill, XII.[19]48, leg. van Someren; 1 female (BMNH), Mombasa, Kilifi, I.[19]42; Tanzania • 1 male (BMNH), Morogoro, 12.I.[19]23.

*Entyposismendax* Péringuey, 1904. Kenya • 1 male and 3 females (RSCV), Taveta, 1100 m, 20.XI.2011, leg. Snížek (Figs 7, 8, 15, 16, 23, 24, 31, 32, 36, 40); 1 male (RSCV), Katutu, Kithioko, 27.XI.1999, leg. Snížek; 2 males and 1 female (RSCV), Voi, XI.1997, leg. Snížek; Tanzania • 6 males and 3 females (BMNH), Maktau, XII.[19]36, leg. MacArthur; 2 males (NMPC), Arusha, Naberera, 8.–13.IV.1997, leg. J. Rolčík (NMPC).

*Entyposis* sp. Somaliland • 1 female (NMPC), Beerato, ca 990 m, 09°21'99"N, 45°03'59"E, 9.–10.VI.2022, leg. David Král & David Sommer (Figs 41–43).

## ﻿Results

### ﻿ Entyposis frici

Taxon classificationAnimaliaColeopteraScarabaeidae

Bezděk & Sehnal sp. nov.

265BCF9C-26FA-5C29-BE59-7291F346114D

https://zoobank.org/AC32231B-E8D5-4308-950D-D6CE53F2A460

Figs 1 , 2 , 9 , 10 , 17 , 18 , 25 , 26 , 33 , 37 , 44

#### Type locality.

Somaliland, Laascaanood [= Las Anod], 8°29.0535'N, 47°22.6342'E, ca 680 m a.s.l.

#### Type material.

***Holotype***, male: “Somaliland, 7.-8.x.2021 | LAAS CANOOD [= Las Anod] – Hamdi Hotel | 8.484225N, 47.377236E | ca 680m, Z. Fric lgt.”. ***Paratype***, female, same data as for holotype.

#### Type depository.

Holotype and paratype are deposited in NMPC.

#### Description of the holotype.

**Figures 1–4.**
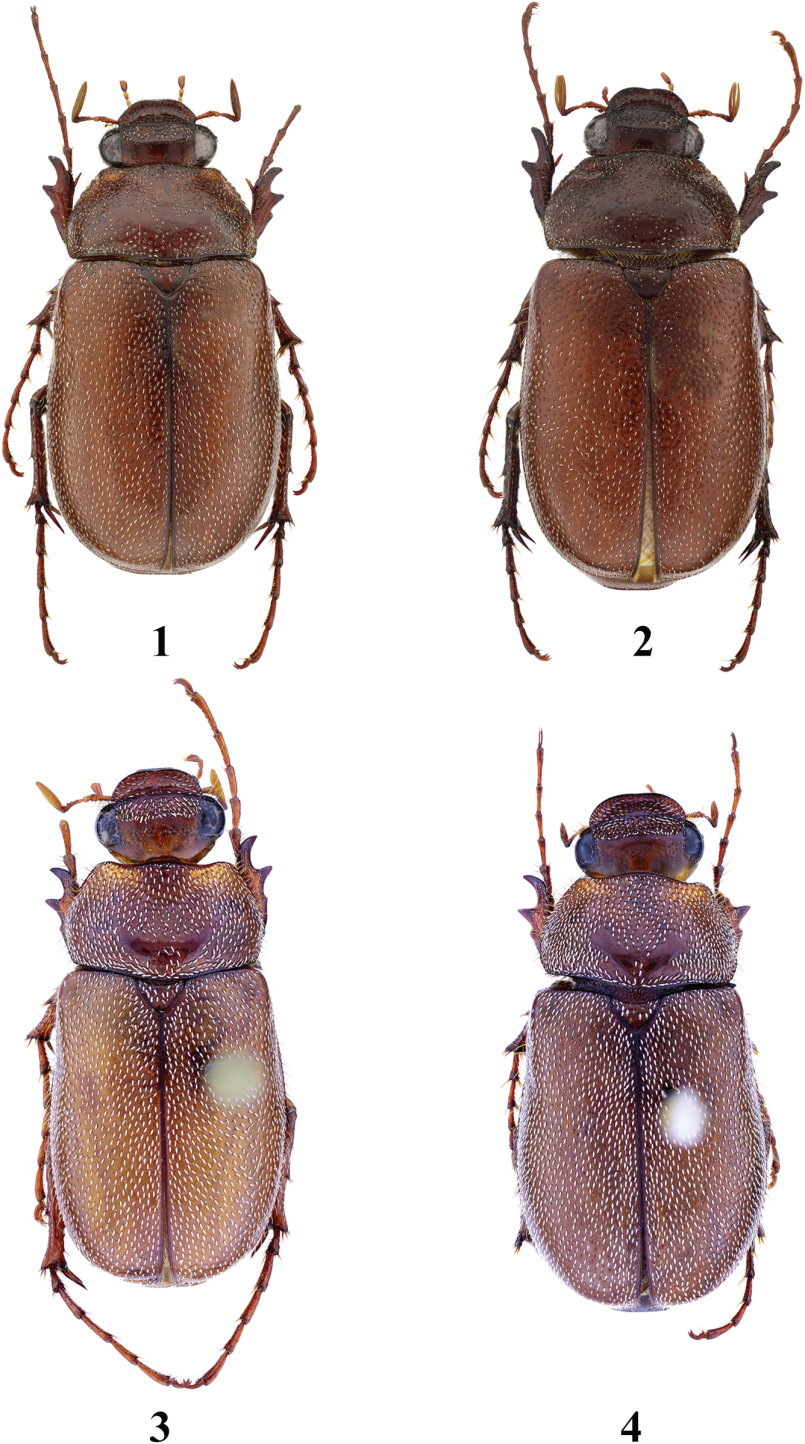
Habitus of *Entyposis* species, dorsal view **1***E.frici* sp. nov., holotype, male (body length 13.6 mm) **2** the same, paratype, female (body length 13.3 mm) **3***E.cordipenis* Sehnal, 2017, paratype, male (body length 13.5 mm) **4** the same, paratype, female (body length 14.2 mm).

**Male.** Body length 13.6 mm (Fig. 1). Body elongate, strongly convex, surface brown, appendages somewhat lighter, moderately shiny, setation pale. Head and pronotum covered with short, stout, semierect setae; elytra with nearly scale-like setation. Legs and ventral surface with sparse, long, erect setae mixed with much shorter, scale-like setae.

Head, including clypeus, densely coarsely punctate, each puncture with short, stout, semierect seta. Clypeus trasverse, broadly rounded, emarginate at middle (Fig. 9). Frontoclypeal carina broadly arcuate. Occipital carina present, prominent. Eye canthus narrow, long, with sparse, long setation. Eye large, distincly extended beyond canthus. Antenna with 10 antennomeres, club trimerous, slightly shorter than antennal shaft. Antennomere 1 with posterior longitudinal row of erect setae and few isolated erect setae at apex; antennomere 2 bare; antennomeres 3–7 with very few (1–4) isolated setae; club sparsely covered with moderately long, erect setae. Labrum transverse, deeply bilobed; lobes rounded, with coarse irregular punctures bearing long, erect setae. Terminal maxillary palpomere narrow, only slightly expanded apically (Fig. 37), distinctly shorter than palpomeres 1 and 2 combined.

Pronotum transverse, convex, widest at about middle; base broader than anterior margin; with shallow, oval anteromedial depression narrower then head weakly rising toward edge of crest (Figs 17, 25). Anterior margin with well visible tubercle at middle; anterior angles weak, broadly rounded. Lateral margins crenulate, with moderately long setae. Posterior margin with distinct border, very shortly interrupted at middle. Crest delimiting anteromedial depression well visible, with wide V-shaped impunctate strip (Fig. 17) and small impunctate area in basal part of depression. Punctation of remaining surface coarse, somewhat irregular, punctures separated by 1–2× their diameters. Each puncture bearing with short, stout, semirecumbent seta.

Scutellum broadly triangular, sparsely and coarsely punctate in basal half, with short, stout setae; apical area impunctate and bare.

Elytron convex, widest at about middle, sutural angle obtuse-angulate. Striae absent; humeral bones present, weakly swolen, impunctate. Surface of elytron moderately shiny, covered with shallow, regularly spaced punctures, separated by 2–3× puncture diameters. Each puncture bearing with short, recumbent, scale-like seta. Epipleuron distinct, complete, narrow, with row of moderately long erect setae. Apical half of lateral margin of elytron with membranous border.

**Figures 5–8.**
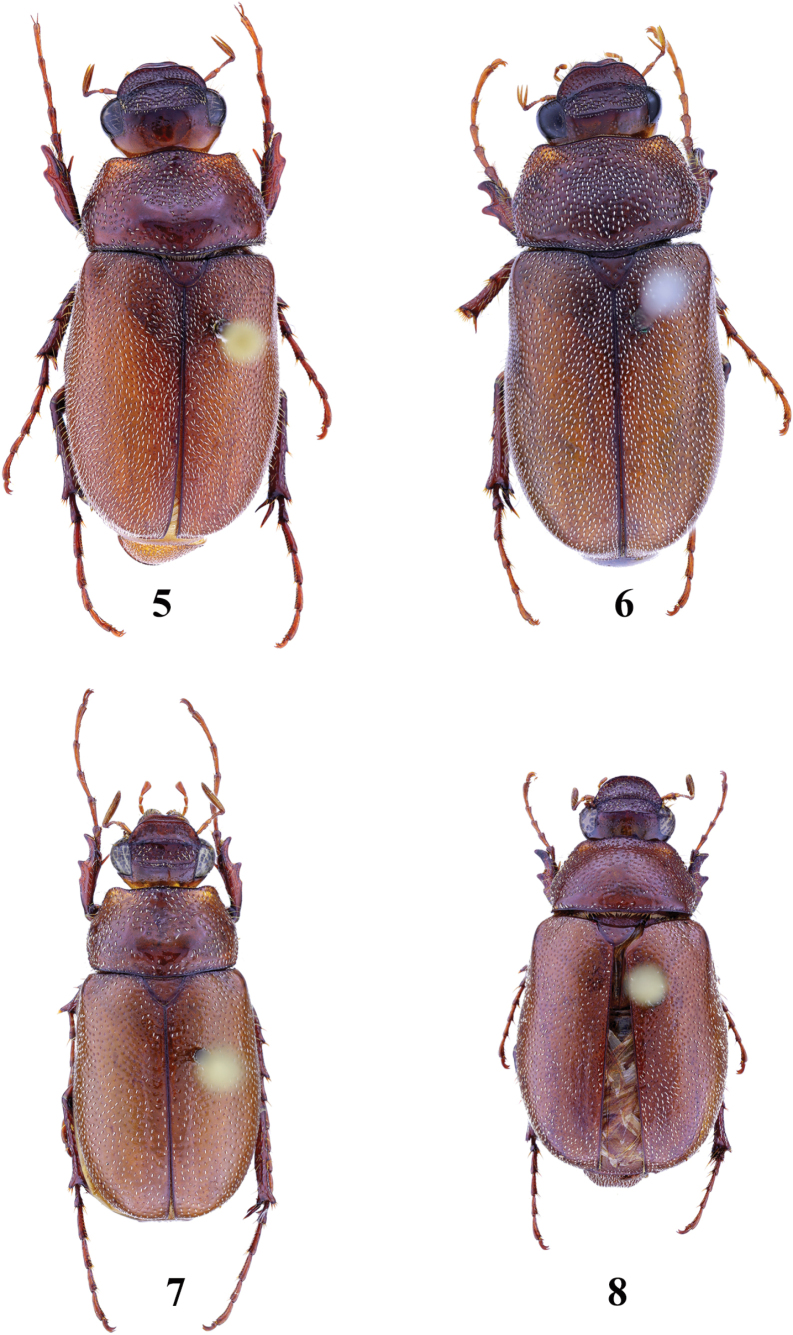
Habitus of *Entyposis* species, dorsal view **5***E.impressa* Kolbe, 1894, male (body length 15.0 mm) **6** the same, female (body length 16.0 mm) **7***E.mendax* Péringuey, 1904, male (body length 11.5 mm) **8** the same, female (body length 10.1 mm).

**Figures 9–16.**
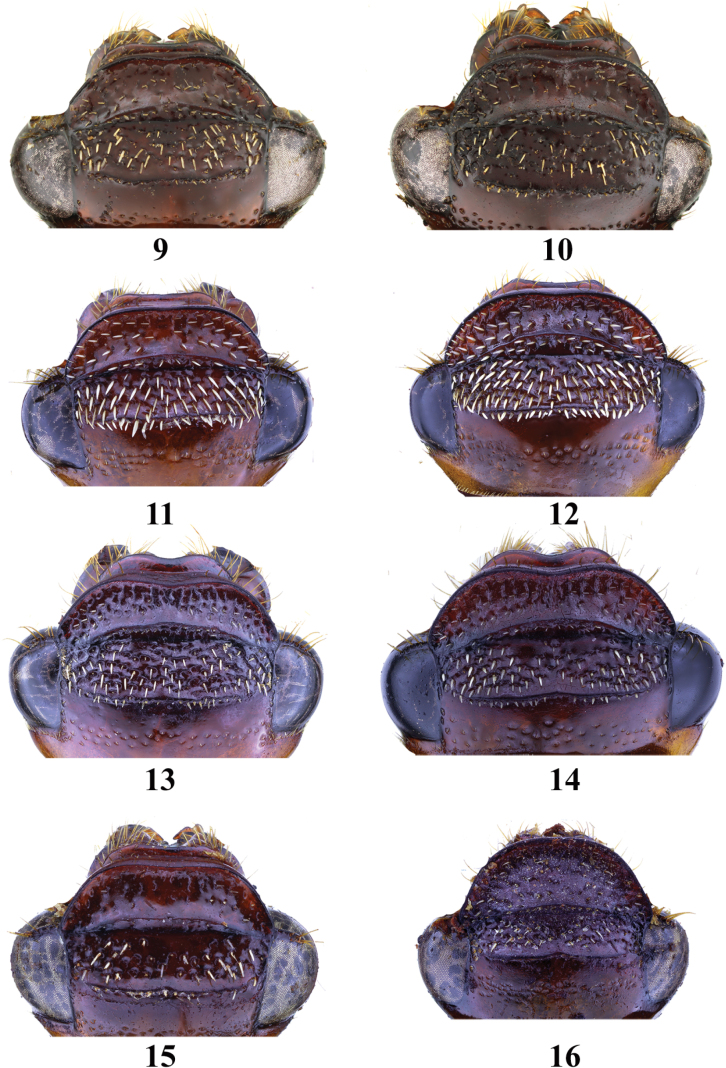
Head of *Entyposis* species, dorsal view **9***E.frici* sp. nov., holotype, male **10** the same, paratype, female **11***E.cordipenis* Sehnal, 2017, male **12** the same, female **13***E.impressa* Kolbe, 1894, male **14** the same, female **15***E.mendax* Péringuey, 1904, male **16** the same, female. Not to scale.

**Figures 17–24.**
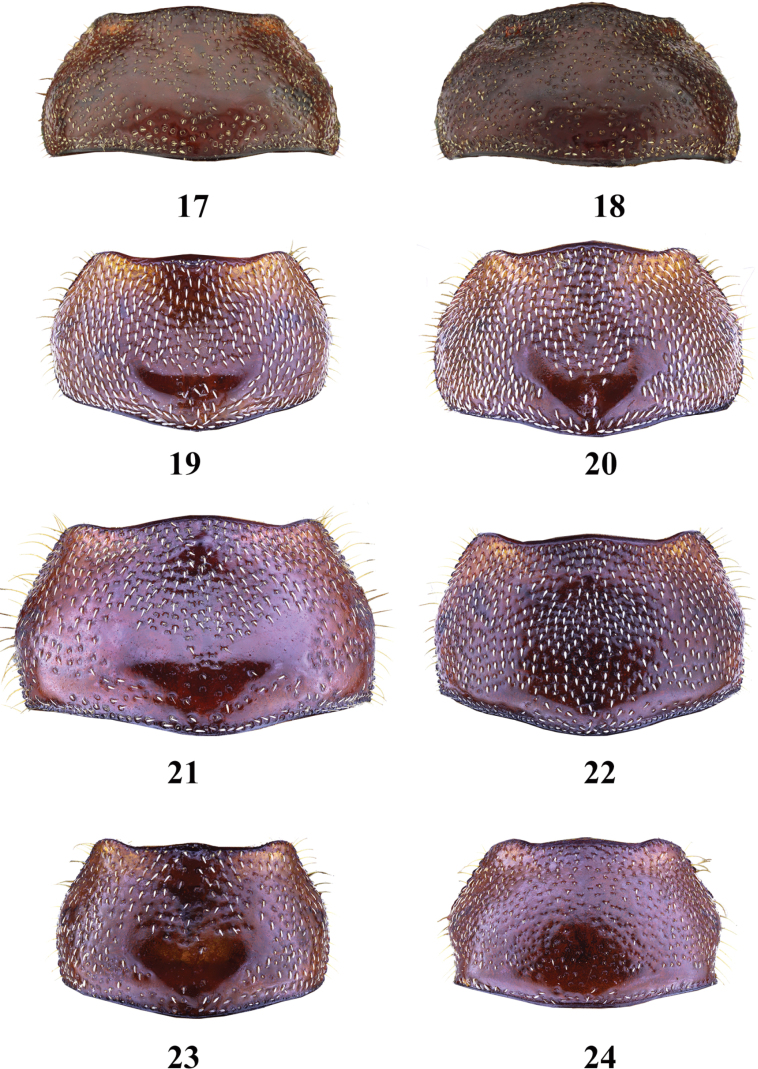
Pronotum of *Entyposis* species, dorsal view **17***E.frici* sp. nov., holotype, male **18** the same, paratype, female **19***E.cordipenis* Sehnal, 2017, male **20** the same, female **21***E.impressa* Kolbe, 1894, male **22** the same, female **23***E.mendax* Péringuey, 1904, male **24** the same, female. Not to scale.

**Figures 25–32.**
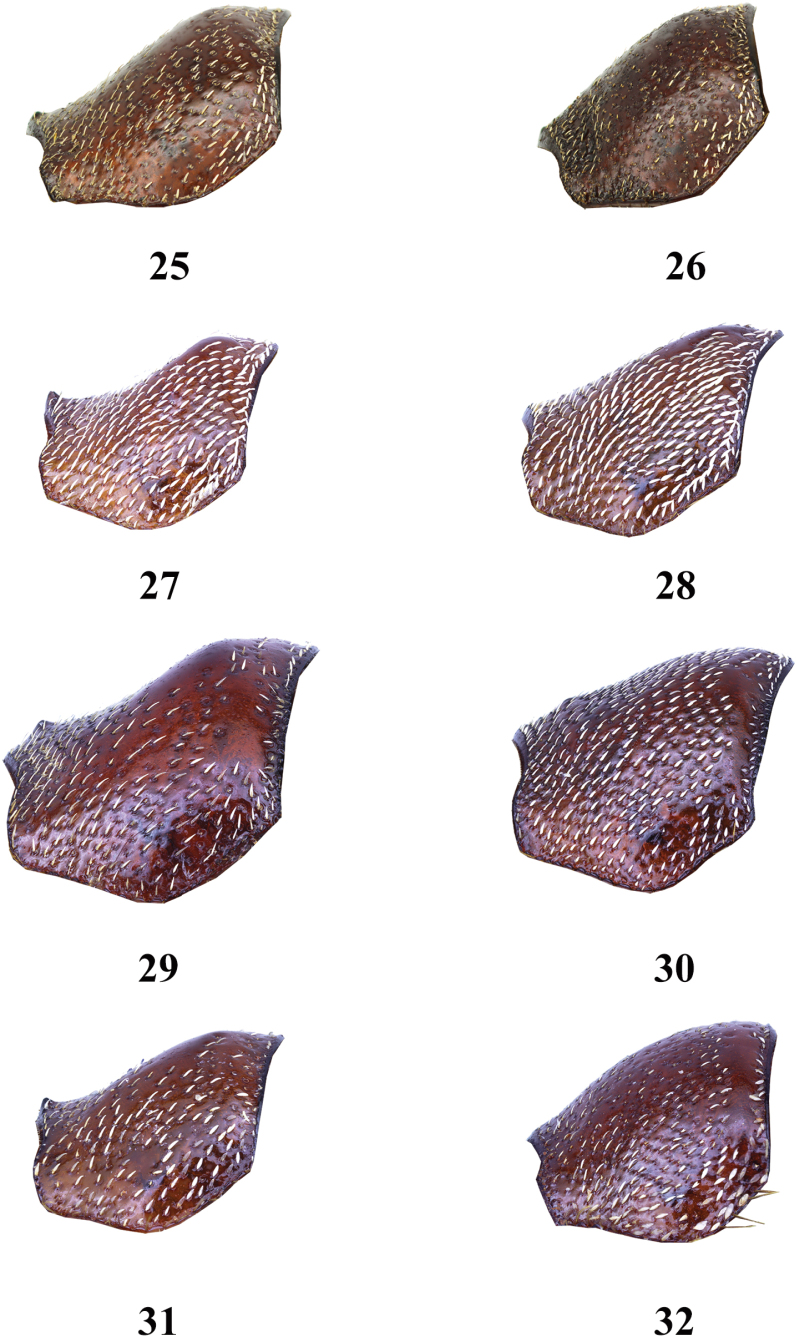
Pronotum of *Entyposis* species, lateral view **25***E.frici* sp. nov., holotype, male **26** the same, paratype, female **27***E.cordipenis* Sehnal, 2017, male **28** the same, female **29***E.impressa* Kolbe, 1894, male **30** the same, female **31***E.mendax* Péringuey, 1904, male **32** the same, female. Not to scale.

Macropterous.

Legs with femora shiny, irregularly punctate, setaceous; setae of metafemora short, partially scale-like. Protibia narrow, distinctly tridentate; terminal spur present, slightly curved externally, acute apically, basal third serrate, inserted against emargination between medial and apical teeth. Mesotibia and metatibia slightly expanded apically, each with one setiferous transversal carina. Mesotibial terminal spurs subequal in length, flattened, acute apically. Upper terminal spur of metatibia flattened, slightly curved, acute apically, about ¼ longer than lower, apically truncate spur. Tarsomeres narrow, long, with two rows of short erect setae ventrally. Each metatarsomere with longitudinal finely serrate crest ventrally. Claws bifid, with vetrobasal teeth and entire ventral edge of lower claw finely serrate.

Ventral surface of thorax sparsely covered with mixture of moderately long erect setae and recumbent scale-like setae. Abdominal ventrites 3–7 covered with irregular punctures bearing recumbent scale-like setae and few isolated moderately long erect setae. Pygidium large, convex, irregularly punctate with short semirecumbent setae. Lateral and apical margins of pygidium distinctly bordered.

**Figures 33–36.**
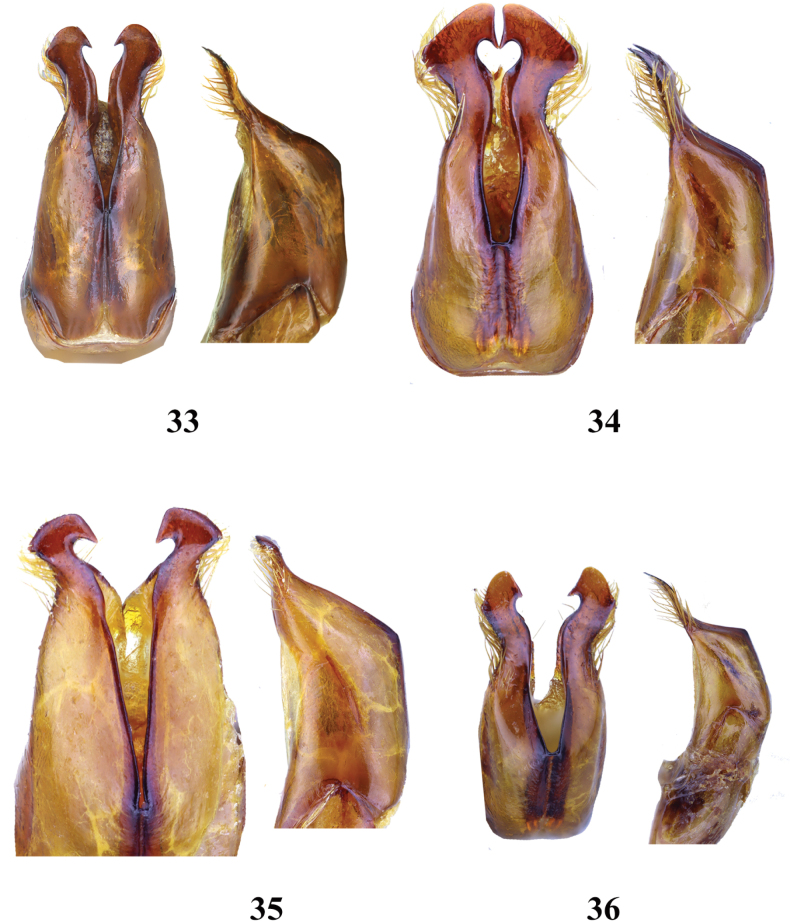
Aedeagus of *Entyposis* species, dorsal and lateral views **33***E.frici* sp. nov., holotype **34***E.cordipenis* Sehnal, 2017 **35***E.impressa* Kolbe, 1894 **36***E.mendax* Péringuey, 1904. Not to scale.

Male genitalia. Parameres symmetrical (Fig. 33), fused basally, longer than phalobase.

**Female** (Figs 2, 10, 18, 26) differs from male in the following characters: body length 13.3 mm, antennal cbub distinctly shorter then antennomeres 1–7 combined, pronotal anteromedial depression less developed, tarsomeres of all legs shorter.

#### Differential diagnosis.

*Entyposisfrici* sp. nov. belongs to a group of species with a shallow anteromedial depression. In the key of the genus *Entyposis* (Sehnal 2017), *E.frici* sp. nov. keys to the couplet with *E.cordipenis*. The male of *E.frici* sp. nov. differs from those of *E.cordipenis* in the shape of the pronotum—the anteromedial depression is only very faintly visible in the lateral view in *E.frici* sp. nov. (Fig. 25), whereas it is more pronounced in *E.cordipenis* (Fig. 27). The male genitalia of the two species are different, in *E.cordipenis* the apical part of the paramere (in dorsal view) is broad (Fig. 34), whereas in *E.frici* sp. nov. it is narrow (Fig. 33).

**Figures 37–40.**
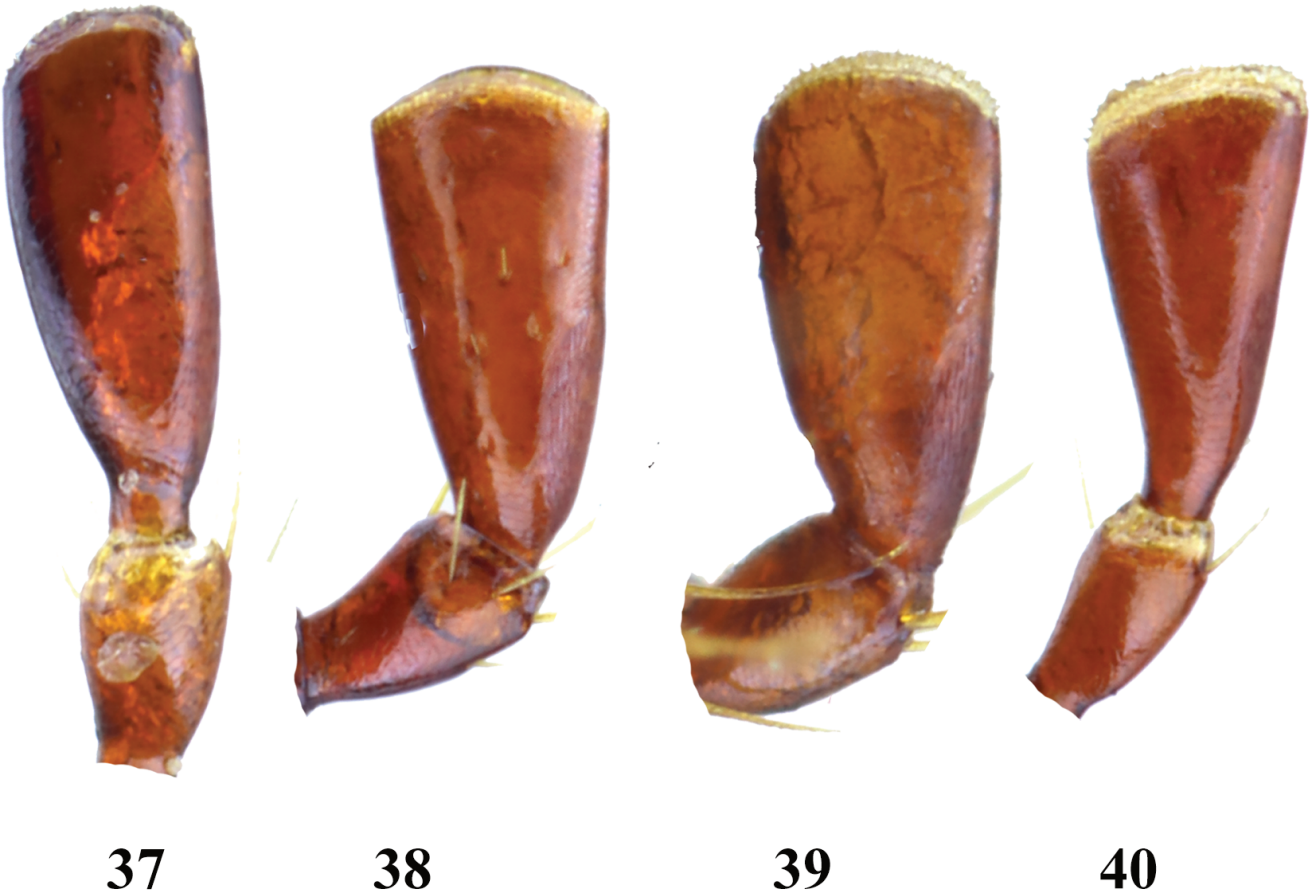
Terminal maxillary palpomere of *Entyposis* species **37***E.frici* sp. nov. **38***E.cordipenis* Sehnal, 2017 **39***E.impressa* Kolbe, 1894 **40***E.mendax* Péringuey, 1904. Not to scale.

Three additional *Entyposis* species are known from Kenya and northern Tanzania, fairly close to the Horn of Africa: *E.impressa*, *E.mendax*, and *E.squamulata*. *Entyposisimpressa* and *E.mendax* share a shallow anteromedial depression with *E.frici* sp. nov. *Entyposisimpressa* is easily distinguished from *E.frici* sp. nov. by the shape of the male genitalia in both dorsal and lateral views (compare Figs 33 and 35). The differences in the shape of the male genitalia between *E.frici* sp. nov. and *E.mendax* are less pronounced (Figs 33 and 36), but both species differ markedly in the shape of the terminal maxillary palpomere, which is elongate in *E.frici* sp. nov. (Fig. 37), but shorter and apically expanded in *E.mendax* (Fig. 40).

No specimen of *E.squamulata* is available to us. This species was described rather recently based on two males only. According to the primary description (Lacroix and Montreuil 2012), males of *E.squamulata* are clearly distinguished from *E.frici* sp. nov. by a distinct anteromedial depression and by the shape of the tubercle at the middle of the anterior margin of the pronotum, which is bilobed in *E.squamulata* but simple in *E.frici* sp. nov.

#### Collecting events.

Both specimens were attracted by UV light trap and sat on the wall of the Hamdi Hotel (Zdeněk Faltýnek Fric pers. comm. 2022).

#### Etymology.

The name of the species is dedicated to Zdeněk Faltýnek Fric, a specialist in the phylogeny and ecology of butterflies, the collector of the type series.

#### Distribution.

The species is known only from the type locality, Laascaanood, Somaliland (Fig. 40).

#### Remark.

**Figures 41–43.**
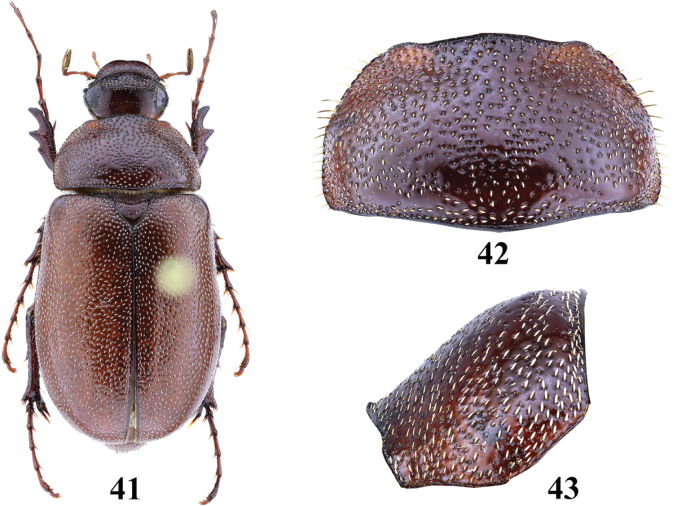
Female of probably undescribed *Entyposis* species from Beerato (Somaliland) (length 15.0 mm) **41** habitus, dorsal view **42** pronotum, dorsal view **43** pronotum, lateral view. Not to scale.

**Figure 44.**
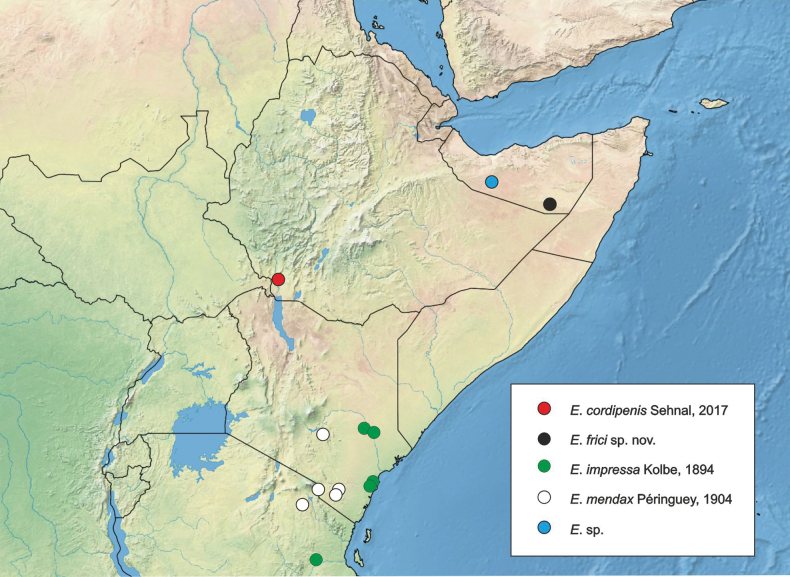
Geographic distribution of *Entyposis* species in the Horn of Africa based on specimens examined.

Members of *Entyposis* seem to be rarely collected. Most species are known from a very few specimens, even only from the type series. We have examined about 500 specimens of Schizonychini collected by Czech entomologists in Somaliland during the last four years. Among this rich material, only three specimens of *Entyposis* were found. In addition to *E.frici* sp. nov. we discovered a single female of probably undescribed *Entyposis* from Beerato (Figs 41–44). We have postponed its description until more specimens, including males, are available.

##### ﻿Key to the species of *Entyposis* from northeastern Africa, males only

**Table:** 

1	Tubercle at middle of anterior margin of pronotum bilobed	***E.squamulata* Lacroix & Montreuil, 2012**
–	Tubercle at middle of anterior margin of pronotum simple	**2**
2	Anteromedial depression of pronotum shalow and very faintly visible in lateral view (Figs 25, 29, 31)	**3**
–	Anteromedial depression of pronotum more distinctly pronounced in lateral view (Fig. 27)	***E.cordipenis* Sehnal, 2017**
3	Narrow tip of male paramera (lateral view) very short (Fig. 35)	***E.impressa* Kolbe, 1894**
–	Narrow tip of male paramera (lateral view) distinctly longer (Figs 33, 36)	**4**
4	Terminal maxillary palpomere elongate (Fig. 37)	***E.frici* sp. nov.**
–	Terminal maxillary palpomere expanded apically (Fig. 40)	***E.mendax* Péringuey, 1904**

##### ﻿Checklist of the genus *Entyposis*

***Entyposis* Kolbe, 1894** (type species *Schizonychacavicollis* Fairmaire, 1887; subsequent designation by Lacroix and Montreuil 2012)

= *Proseconius* Kolbe, 1894 (type species *Schizonychacapito* Gerstaecker, 1873; monotypy); synonymized by Sehnal (2019).

***Entyposisbidentata* Lacroix & Montreuil, 2012**

**Distribution.** Mozambique (Lacroix and Montreuil 2012).

***Entyposiscapito* (Gerstaecker, 1873)**

= *Schizonychacavicollis* Fairmaire, 1887; synonymized by Sehnal (2019).

**Distribution.** Tanzania, including Zanzibar Island (Lacroix and Montreuil 2012; Sehnal 2019).

***Entyposiscordipenis* Sehnal, 2017**

**Distribution.** Southern Ethiopia (Sehnal 2017).

***Entyposisexcavata* Lacroix & Montreuil, 2012**

**Distribution.** Tanzania (Lacroix and Montreuil 2012).

***Entyposisfrici* Bezděk & Sehnal, sp. nov.**

**Distribution.** Somaliland (this paper).

***Entyposisimpressa* Kolbe, 1894**

**Distribution.** Kenya, Tanzania (Lacroix and Montreuil 2012).

***Entyposismadogolelei* Lacroix & Montreuil, 2012**

**Distribution.** Mozambique (Lacroix and Montreuil 2012).

***Entyposismartinezi* Lacroix & Montreuil, 2012**

**Distribution.** Mozambique (Lacroix and Montreuil 2012).

***Entyposismendax* Péringuey, 1904**

= *Schizonychanyukana* Kolbe, 1910; synonymized by Lacroix and Montreuil (2012).

= *Entyposismontana* Moser, 1913; synonymized by Lacroix and Montreuil (2012).

**Distribution.** Kenya, Tanzania, Zimbabwe (Lacroix and Montreuil 2012).

***Entyposisrasplusi* Lacroix & Montreuil, 2012**

**Distribution.** Mozambique (Lacroix and Montreuil 2012).

***Entyposissquamulata* Lacroix & Montreuil, 2012**

**Distribution.** Kenya, Tanzania (Lacroix and Montreuil 2012).

## Supplementary Material

XML Treatment for Entyposis frici

## ﻿Additional information

### Author ORCIDs

Aleš Bezděk https://orcid.org/0000-0002-8748-3281

Richard Sehnal https://orcid.org/0000-0002-3262-9132

Hassan S. A. Elmi https://orcid.org/0000-0002-1810-8170

David Sommer https://orcid.org/0000-0003-4519-9677

David Král http://orcid.org/0000-0002-1439-7892

### Data availability

All of the data that support the findings of this study are available in the main text or Supplementary Information.
